# Psychometric Analysis of the Shortened Version of the Spiritual Well-Being Scale on the Slovak Population (SWBS-Sk)

**DOI:** 10.3390/ijerph19010511

**Published:** 2022-01-03

**Authors:** Peter Tavel, Bibiana Jozefiakova, Peter Telicak, Jana Furstova, Michal Puza, Natalia Kascakova

**Affiliations:** 1 Olomouc University Social Health Institute, Palacky University Olomouc, Univerzitní 22, 77111 Olomouc, Czech Republic; peter.tavel@oushi.upol.cz (P.T.); jana.furstova@oushi.upol.cz (J.F.); michal.puza@oushi.upol.cz (M.P.); natalia.kascakova@oushi.upol.cz (N.K.); 2 Institute of Experimental Psychology, Centre for Social and Psychological Sciences, Slovak Academy of Sciences, Dúbravská Cesta 9, 84104 Bratislava, Slovakia; peter.telicak@savba.sk; 3 Psychiatric-Psychotherapeutic Outpatient Clinic, Heydukova 27, 81108 Bratislava, Slovakia

**Keywords:** spiritual well-being, SWBS, Slovak, representative sample, validation

## Abstract

This study was focused on verifying the factor structure of the shortened version of the Spiritual Well-Being Scale (SWBS) on a representative sample of adult Slovak citizens (*N* = 1018, 49% men, age 18–85 years, and mean age 46.2). The shortened version of the SWBS consists of 10 items divided into two subscales: religious well-being (RWB) and existential well-being (EWB). Results of confirmatory factor analysis (CFA) showed insufficient parameters of the full two-factor model due to three negatively formulated items. After their exclusion, the two-factor model was found to be valid in the Slovakian population (χ (13) = 53.1, *p* < 0.001, χ^2^/df = 4.1, CFI = 0.999, TLI = 0.999, RMSEA = 0.055, and SRMR = 0.028). The reliability of the final version of the SWBS-Sk, consisting of seven positively worded items, is high, with α = 0.86 and ω = 0.94. Religious respondents and women scored significantly higher on the whole scale (*p* = 0.001) as well as on the two subscales (*p* < 0.05). A higher age was associated with a higher RWB score (*p* = 0.001) and a lower EWB score (*p* = 0.002). The shortened version of the SWBS-Sk consisting of positively worded items was found to be valid and reliable for further use in the Slovak environment.

## 1. Introduction

Spirituality and religiosity are considered the sources of a meaningful life [1,2,3]. The terms “spirituality” and “religiosity” tend to be confused in common speech because of their seemingly similar meaning and [4] because these terms have a range parallel with the fact that religiosity is the public side of spirituality, manifested through traditions and institutions. Religiosity provides a set of beliefs, goals, and meanings that can help solve personal situations and problems [3]. Spirituality is considered a multidimensional construct that exceeds religiosity. It includes the terms of subjective well-being, the meaning of life or harmony [5].

The importance of spirituality and religiosity for mental health has been validated in adults [6,7] and also in adolescent populations [8]. Simultaneously, spirituality may serve as an efficient coping strategy for people who are in a difficult life situation, such as the loss of a spouse [9], or who have survived potentially traumatic life events, such as a natural disaster [10,11,12]. For these reasons, it is important to have an effective method of measuring spirituality and its overlap with well-being. A commonly used tool for measuring subjective well-being in its holistic, i.e., spiritual and existential, meaning is the Spiritual Well-Being Scale (SWBS) created by Paloutzian and Ellison [13]. Research using the SWBS predominantly has focused on assessing its associations with different aspects of health, e.g., depression [14,15,16], suicidality [17,18], or psychopathology in general [19].

The SWBS has been used in over 300 publications and has been translated into 10 different languages. When verifying the factor structure of the questionnaire across different translations and cultures, authors have mainly validated the original two-factor structure comprising two dimensions, religious well-being (RWB, vertical dimension) and existential well-being (EWB, horizontal dimension). Besides the differences related to various language and cultural adaptations, it seems that the factor structure varies across different samples. For example, Musa and Pevalin [15] found a two-factor structure in their study of hemodialysis patients in Jordan, while Scott, Agresti, and Fitchett [20] reported a three-factor structure in a sample of psychiatric patients. A three-factor structure was also reported in a sample of patients with acute myocardial infarction [21]. Moreover, the factor structure of the SWBS also varied across different samples of college students, with a two-factor structure in a Jordanian Arab group and a three-factor structure in groups of Malaysian students [22] and Portuguese students [23].

Most of the previously reported research has used the full 20-item version of the questionnaire. However, Cotton et al. [8] proposed a shortened, 10-item, version of the SWBS questionnaire, which is, due to its briefness, more convenient for use in large test batteries. This shortened version was validated by Malinakova et al. [24], who suggested an adjusted 7-item version of the SWBS, consisting only of positively worded items. Consequently, this adjusted version has been used in other studies [25,26]. Other studies focusing on the psychometric analysis of the full version of the SWBS on a representative sample of Czech adults [27] and Turkish respondents [28] have reported the same conclusion, i.e., a recommendation to exclude the negatively worded items.

Therefore, for the reasons of (1) potential cultural differences in responses to the items and (2) the fact that the validity of the original version of the questionnaire does not guarantee its validity in other languages [29], the main goal of this study was to verify the psychometric properties of the shortened Slovak version of the SWBS. An additional goal of the study was to compare sociodemographic groups in terms of their spiritual well-being.

## 2. Materials and Methods

### 2.1. Participants and Procedure

The sample in the current study was collected in a cross-sectional design. Data were collected in April 2019 through a professional survey agency on behalf of the research team. The data were collected by professionally trained administrators using the method of computer-assisted personal interviewing (CAPI) [30,31]. CAPI is a face-to-face data collection method in which the interviewer uses a tablet or a computer to record answers given during the interview. The advantage of this collection method is that it allows interviews of longer duration with a more complex questionnaire design (which was the case in our study). It eliminates errors in recording answers and significantly saves time by speeding up the processing of the collected data [30].

A preliminary test on 10 respondents of different genders, ages, and education levels was performed prior to the study with the aim of checking the reliability of the questionnaire. Then, respondents from a list of inhabitants of the Slovak Republic, stratified by gender, age, education, size of place of living, and region of living, were contacted via telephone by administrators and asked to participate in a larger study on health. They were informed about the expected duration (approximately 45–60 min), confidentiality and protection rules, and the right to decline to participate and withdraw from the research.

The final sample consisted of 1018 (522 women and 496 men) respondents forming a representative sample of the Slovak adult population. Quota characteristics of the sample (gender, age, education, size of place of living, and region of living) were calculated based on data from the Statistical Office of the Slovak Republic [32]. Participation in the survey was voluntary and anonymous. Respondents agreed with the electronic informed consent, including the data protection declaration, before their participation in the study. The study was conducted at the participants’ homes. All procedures were carried out according to the ethical standards of the institutional and national research committee and the Declaration of Helsinki. The study was approved by the Ethics Committee of the Olomouc University Social Health Institute, Palacky University Olomouc (No. 2019/05).

### 2.2. Measures

The Spiritual Well-Being Scale (SWBS) [13] is a self-reported questionnaire that measures spiritual well-being in terms of its holistic meaning [33]. The original version consists of 20 items. For the purpose of this study, which comprised a larger battery of questionnaires for covering more variables, a shortened version of the SWBS [8] was used as a suitable tool for a quick screening of spiritual well-being. The translation of the shortened Slovak version of the SWBS was obtained by forward translation. It consists of 10 items, which can be answered on a 6-point Likert scale, ranging from 1 = strongly disagree to 6 = strongly agree. Theoretically, the SWBS contains two subscales: (1) religious well-being (RWB), focusing on subjective well-being concerning God, and (2) existential well-being (EWB), focusing on the meaning of life and general satisfaction with life. Each subscale contains 5 items. Three of them (item no. 5 in the RWB and items no. 1 and 8 in the EWB) are scored reversibly. The total SWBS-Sk score varies from 10 to 60.

The Brief Resilience Scale (BRS) [34] consists of 6 items assessed on a 5-point Likert scale, ranging from 1 = strongly disagree to 5 = strongly agree. The BRS measures resilience as the ability to recover or “bounce back” from a stressful life event. The Slovak version of the BRS used in a previous study has shown good psychometric properties, validity, and reliability [35].

The Post-traumatic Growth Inventory (PTGI) [36] measures the level of post-traumatic growth in persons who have survived a traumatic event. It consists of 21 items, which can be answered on a 6-point Likert scale, ranging from 0 = “I did not experience this change as a result of my crisis” to 5 = “I experienced this change to a very great degree as a result of my crisis.” The questionnaire contains five subscales: (1) relating to others, (2) new opportunities, (3) personal strength, (4) spiritual change, and (5) understanding of life.

The SF-8 Health Survey [37] consists of 8 items, each of which falls under one subscale: (1) general health (GH), (2) physical functioning (PH), (3) role physical (RP), (4) bodily pain (BP), (5) vitality (VT), (6) social functioning (SF), (7) mental health (MH), and (8) role emotional (RE). The outcome may be summarized into a physical component summary (PCS) as a total physical health score and a mental component summary (MCS) as a total mental health score.

Demographics: The respondents were asked to report their age, gender, marital status, education, and economic activity.

Religiosity: The participants were asked the question “At present, would you call yourself a believer?” and were instructed to choose one of the following options: “Yes, I am a member of a church or religious society,” “Yes, but I am not a member of a church or religious society,” and “No”—“No, I am a convinced atheist”.

### 2.3. Statistical Analyses

All the statistical analyses were performed using the R software, version 4.0.5 [38]. R is among the most widely used statistical software in the world. It is used by researchers from diverse disciplines. It is downloadable online and has incredible flexibility and power to perform statistical computing. Confirmatory factor analysis (CFA) was used to assess the factor structure of the SWBS-Sk. CFA was calculated using the Lavaan package [39] with diagonally weighted least squares (DWLS) estimation of the parameters. Lavaan is a standard R library for SEM computing; it contains all the functions necessary for fitting a model and testing its performance. A matrix of polychoric correlation served as the basis for the computations. Several measures were examined as fitting the parameters of CFA models: The comparative fit index (CFI), the Tucker–Lewis index (TLI), root-mean-square error of approximation (RMSEA), and the standardized root-mean-square residual (SRMR). In concordance with Hu and Bentler [40], the values of CFI and TLI > 0.95, RMSEA < 0.06, and SRMR < 0.08 were considered an excellent fit. A set of scaled chi-square difference tests [41] was executed to determine the best-fitting model. Cronbach’s alpha and McDonald’s omega coefficients were evaluated for the reliability assessment of the SWBS-Sk. Histograms and the Shapiro–Wilk test were used to verify normal distribution of the data. The assumption of normality was not met in the data; therefore, nonparametric statistical methods were used for further analyses. Convergent validity of the SWBS was assessed with the nonparametric Spearman correlation coefficients. The measurement invariance of the final CFA model was tested to ascertain whether the SWBS-Sk means could be compared between sociodemographic groups. Nonparametric tests were employed for comparison: the Mann–Whitney U test for the comparison of two groups and the Kruskal–Wallis test with Dunn–Bonferroni correction for multiple group comparison. The significance level was set at *p* < 0.05 for all statistical significance testing. In addition to the *p*-values, Cohen’s d effect size coefficients were evaluated. The delta sign (Δ) is applied as a label for the “difference” throughout the Results section, and it is always used in the absolute (positive) value.

## 3. Results

### 3.1. Construct Validity

Table 1 shows item analysis and correlations of the items with the whole scale. Correlations of the positively formulated items range from r = 0.46 to r = 0.67. The negatively formulated items, however, show low correlation with the scale, with values below 0.30.

**Figure 1:**
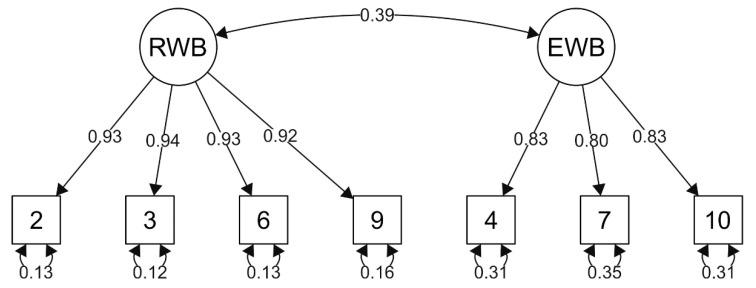
The best-fitting CFA model (Model 3): the two-factor model of the SWBS-Sk with positively worded items only. RWB: Religious Well-Being; EWB: Existential Well-Being.

Three models supported in previous studies [24,27,42,43,44] were examined in the current study: first, a one-factor model that includes all the items (Model 1); then a two-factor model with items separated into two factors, the Religious Well-Being (RWB) and Existential Well-Being (EWB) subscales (Model 2); and finally, a two-factor structure including only positive-worded items (Model 3). A second-order hierarchical model and a bifactor model could not be tested because in these models, the covariance matrices of latent variables were not positively definite and thus the standard errors could not be computed. As presented in Table 2, the criteria values of CFI, TLI, SRMR, and RMSEA show that the one-factor and two-factor models containing all items (Models 1 and 2) do not have a satisfactory fit to the data. The only model with satisfactory criteria values was the two-factor model with positive-worded items only (Model 3). The factor loadings of the positive items in all three tested models were high, with values above 0.60. On the contrary, the factor loadings of negative-worded items in Models 1 and 2 were low, with values ranging from 0.20 to 0.50. In Model 3, the loadings of all items were high, with values of 0.92–0.94 in the RWB subscale and 0.80–0.83 in the EWB subscale (see Figure 1).

The correlation between the RWB and EWB subscales is r = 0.39. Given the above information, the two-factor model with positive items only (Model 3) was evaluated as the best fit to the data. Therefore, the reliability, validity, and group differences were analyzed for this model only. From now on, we use the abbreviation SWBS-Sk to indicate the shortened Slovak version of the scale consisting of seven positively worded items.

### 3.2. Reliability

The internal consistency of the shortened SWBS-Sk scale (Model 3) was measured using Cronbach’s α and McDonald’s ω coefficients. Both coefficients showed the high reliability of the scale, with α = 0.86 (95% CI 0.85–0.87) and ω = 0.94. The reliability of the RWB subscale with positive items was α = 0.95 (95% CI 0.94–0.95) and ω = 0.96; the reliability of the EWB subscale with positive items was α = 0.81 (95% CI 0.79–0.83) and ω = 0.81.

### 3.3. Convergent Validity

Correlations between spiritual well-being (SWBS-Sk), religious well-being (RWB), existential well-being (EWB), resilience (BRS), post-traumatic growth (PTGI), and health-related well-being (SF-8) were used to assess the convergent validity of the SWBS-Sk and its subscales (see Table 3). SWBS-Sk, RWB, and EWB were positively correlated with all subscales of the PTGI. The strongest correlations were found between the PTGI subscale “Spiritual Change” and the summary score of SWBS-Sk and RWB (rho = 0.45, resp. rho = 0.502, and *p* < 0.001). The EWB subscale was positively correlated with the BRS (rho = 0.17 and *p* < 0.001), PCS, and MCS (rho = 0.26, resp. rho = 0.32, and *p* < 0.001). The correlation analysis suggests that the SWBS-Sk and its subscales are related to other similar constructs. Even though the correlation coefficients are rather weak, the majority of them are significantly non-zero. Further exploration of the convergent validity of the SWBS in the Slovak environment may be needed.

### 3.4. Differences in SWBS-Sk Scores among Sociodemographic Groups

To assess differences between sociodemographic groups in the SWBS-Sk scores, the measurement invariance of the final two-factor model was tested. Strong measurement invariance was found for gender (Δχ^2^(26) = 30.40, *p* = 0.251, ΔCFI < 0.001, and ΔRMSEA = 0.019) and age groups (Δχ^2^(130) = 136.57, *p* = 0.329, ΔCFI < 0.001, and ΔRMSEA = 0.029). In the case of religiosity, no strong measurement invariance was confirmed. However, the differences in the fit indices ΔCFI and ΔRMSEA suggest the criterion for invariance as presented by Chen [45] has not been violated (Δχ^2^(15) = 27.17, *p* = 0.027, ΔCFI = 0.003, and ΔRMSEA = 0.009). Therefore, the SWBS means between gender, age, and religious groups were compared (see Table 4).

Regarding gender, women scored significantly higher than men on the whole scale as well as on the two subscales (with a small to moderate effect size of d ranging from 0.14 to 0.40). Differences in the age groups were found in the RWB and EWB subscales only. A higher age was associated with a higher RWB score (d = 0.25) and a lower EWB score (d = 0.24). Religious believers showed the highest scores on the total SWBS (with a large effect size of d = 1.61) as well as on its subscales (with a large effect size of d = 1.84 in RWB and a low effect size of d = 0.23 in EWB). The lowest mean score of religious well-being was reported by atheists, while the lowest mean score of existential well-being was reported by non-believers. In this case, “non-believers” are the people who chose the answer “no” when they were asked if they are religious. In other words, they did not see themselves as convinced atheists.

## 4. Discussion

This study aimed at verifying the factor structure of the shortened Slovak version of the Spiritual Well-Being Scale (SWBS) on a representative sample of the Slovak adult population and comparing the sociodemographic groups regarding their spiritual well-being. The analyses revealed low correlations between the negatively formulated items and the total score. The results also suggested that a two-factor model formed by positively formulated items only forms a valid and reliable version of the shortened scale (SWBS-Sk). The final two-factor model was invariant across genders, age groups, and religious groups. We also verified the convergent validity of the SWBS-Sk and its subscales (religious well-being (RWB) and existential well-being (EWB)) with the studied variables, i.e., posttraumatic growth (PTGI), resilience (BRS), and summary scores of physical and mental health (PCS and MCS).

The SWBS is a widely used tool to measure spirituality. The scale has been validated in various populations, for example, in adolescents [24], children [46], and adult populations [24,47]. The questionnaire has also been translated into various languages, such as Greek [48], Czech [27], and French [49]; for a review, see Ai et al. [33]. Since the good psychometric properties of the original questionnaire do not warrant such results in its adaptations, multiple studies have worked with a modified factor structure of the scale [28,48,50]. These differences may occur for a variety of reasons, for example (1) the different statistical methods used to verify the factor structure, e.g., confirmatory factor analysis or exploratory factor analysis, (2) differences in the research samples caused by cultural differences, including diverse concepts of religiosity, or (3) differences caused by positive/negative wording of the items. The latter has evoked extensive discussion among researchers about whether using negatively worded items in scales is appropriate. Such items can be problematic because of their potentially unclear meaning to respondents [51,52], and thus the data can be contaminated by respondents’ inattention and confusion [53], especially in the case of double-negative formulations [54]. Therefore, the exclusion or a priori avoidance of negatively formulated items is a method used by some authors [55,56]. However, simply excluding negatively worded items from a measurement does not make the instrument problem-free, and researchers should be aware of the potential response bias [54].

Our findings of differences in the level of spirituality among women and men are in line with previous research [24,27,57,58,59]. It is relatively common to talk about gender differences in psychology. Some variables, such as emotionality, are known to be mostly attributed to women [60]. However, this does not mean that men lack experiencing emotions. A more considerable manifestation of those feelings may be connected to the cultural environment, where women are more expected to express their feelings or other contextual conditions [61]. Similar cultural differences may also be expressed through the level of spirituality. The items in the SWBS ask about feelings (e.g., “I feel very fulfilled and satisfied with my life”), beliefs (e.g., “I believe that God loves me and cares about me”), and/or inner perception of relationships (e.g., “I have a personally meaningful relationship with God”). Women may find it easier to identify with this form of expressing themselves.

The results of age differences in spirituality or religiosity level are also in line with previous researchers [27,57,62]. The period of getting older is connected with the awareness of reaching the later phase of life. At the same time, it is a period of more frequent somatic diseases and difficulties. This could be the reason why older people become more spiritual and religious [62]. There may also be so-called age cohort differences caused by the education and culture of the era when they grew up [63].

In the present study, believers showed the highest spiritual well-being, both religious and existential. The lowest religious well-being was found in the group of atheists, which is consistent with their attitude toward religion. However, in the case of existential well-being, the lowest score was reported by non-believers, not atheists. This finding is in agreement with Galen and Kloet [64], who described a curvilinear relationship of well-being among believers and non-believers. In their view, people with higher well-being were those more confident in their worldview, both strong believers as well as atheists. Those who were unsure or agnostic had lower levels of well-being. Existential well-being, with its focus on life satisfaction and the meaning of life, thus seems to be less connected with the religious beliefs themselves and more related to the strength of their attitude. Our examination of the convergent validity of the SWBS-Sk showed a positive relationship between spiritual well-being and post-traumatic growth (PTG). PTG is described as positive changes in some areas of life as a result of struggling with trauma [37,65]. One of the areas of growth is spirituality. The relationship between spirituality and PTG was studied by various researchers, e.g., Parappully et al. [66], Werdel et al. [67], and Khursheed and Shahnawaz [68]. It is also in line with the theory of PTG process as described by Tedeschi and Calhoun [37], which describes spiritual change as one of the main aspects of PTG. The connection between spiritual change and PTG was shown to be so strong that it became one of the domains of the Post-traumatic Growth Inventory (PTGI) [69]. In the present study, the results reveal a positive relationship between the total score of the PTGI and the SWBS-Sk subscales, with the strongest relationship between the spiritual change subscale and the total score of the SWBS-Sk and the RWB subscale. PTG is sometimes considered as a concept equivalent to resilience [70]. In reality, however, PTG goes beyond resilience with its actual transformative potential [37].

In our study, there was a positive relationship between resilience as the ability to “bounce back” in stressful situations and existential well-being (the EWB subscale), but not between resilience and religious well-being (the RWB subscale). Such results are consistent with research findings that describe a positive relationship between resilience and life satisfaction [71,72,73], which is a part of existential well-being (EWB).

Our results also point to a positive relationship between physical health (PCS), mental health (MCS), and existential well-being (EWB). Previous studies have described spiritual or religious people as reporting better subjective health and its influence on life satisfaction [74] and better mental health [75]. However, some authors have discussed the associations only between spirituality and life satisfaction or subjective health [76]. Lun and Bond [77] reported national-related outcomes as being significant. In cultures in which the aspect of socialization is relevant for religious faith, its spiritual practice was positively correlated with the subjective well-being of the individual, and vice versa. Results of the present study also show a negative relationship between PCS, MCS, and religious well-being (the RWB subscale). The impact of religiosity/spirituality on mental and physical health has been further studied by, e.g., Unterrainer, Lewis, and Fink [78], who highlighted the ambivalent nature of religiosity/spirituality. In their view, religiosity/spirituality does not always play a positive role in the process of recovery from an illness. For some people, religious/spiritual matters might have an aggravating effect. Well-being seems to be more related to trust in God [79], in life in general, society [80], in the closest persons, or in other people formed in early attachment [81]. Although the primary focus of our study was not to explore religious/spiritual matters or the role of trust in relationship to well-being, we consider these topics to be important for future research and further scientific discourse.

### Strengths and Limitations

The major strength of our study is the fact that its results are based on a representative sample of Slovak citizens. However, this study has several limitations. First of all, the SWBS questionnaire was the part of a larger battery of questionnaires and was placed in the last third of the whole battery. The total time of the interview was approximately 45–60 min. For these reasons, the responses of participants may have been influenced by reduced attention and fatigue. Secondly, there was a lack of similar constructs in the questionnaire battery. These could have been used for a more straightforward construct validity computation. Some items of the SWBS questionnaire had potentially problematic formulations (the so-called double-barreled items) and, as such, might have been confusing for the respondents.

## 5. Conclusions

For a quick assessment of spiritual well-being, the shortened version of the SWBS-Sk questionnaire was shown to be both reliable and valid. The present paper evaluated its psychometric properties and provided descriptive insights obtained from a representative sample of the Slovak adult population. Based on our results, it is recommended to use the two-factor model with positively worded items only.

## Figures and Tables

**Table 1:** Descriptive statistics of SWBS items.

SWBS Items	Mean	SD	Correlation with the Scale ^†^
RWB			
2. I believe that God loves me and cares about me.	4.09	1.69	0.67
3. I have a personally meaningful relationship with God.	3.81	1.71	0.65
5R. I don’t get much personal strength and support from God.	4.07	1.52	0.25
6. I believe that God is concerned about my problems.	3.88	1.66	0.67
9. My relationship with God contributes to my sense of well-being.	3.84	1.64	0.63
EWB			
1R. I don’t know who I am, where I came from, or where I’m going.	4.56	1.53	0.26
4. I feel very fulfilled and satisfied with my life.	4.83	1.18	0.50
7. I feel good about my future.	4.59	1.19	0.46
8R. Life doesn’t have much meaning.	4.71	1.49	0.25
10. I believe there is some real purpose for my life.	4.85	1.18	0.51

Note: SWBS = Spiritual Well-Being Scale; RWB = Religious Well-Being subscale; EWB = Existential Well-Being subscale; R = reverse-coded; SD = standard deviation; ^†^ correlation of the item with the whole scale, corrected for overlapping.

**Table 2:** Parameters of fit of several CFA models on the complete SWBS and on the SWBS-Sk consisting of positively worded items only.

Model Fit Parameters	Model 1: 1-Factor Model with All Items	Model 2: 2-Factor Model with All Items	Model 3: 2-Factor Model Positively Worded Items Only
DWLS Chi-Square (df)	3490.41 (35) ***	698.42 (34) ***	53.07 (13) ***
CFI	0.956	0.992	0.999
TLI	0.943	0.989	0.999
RMSEA (90% CI)	0.312 (0.303–0.320)	0.139 (0.130–0.148)	0.055 (0.040–0.071)
SRMR	0.206	0.098	0.028
Δ Chi-Square (Δ df) ^†^		338.19 (1) ***	638.03 (21) ***

Note: df = degrees of freedom; CI = confidence interval; ^†^ chi-square difference test compared to the previous model; *** *p* < 0.001.

**Table 3:** Nonparametric Spearman correlation coefficients between the SWBS-Sk (positively worded items only), BRS, and PTGI and SF-8 measures.

	SWBS-Sk		RWB		EWB	
	rho	(95% CI)	rho	(95% CI)	rho	(95% CI)
Brief Resilience Scale (BRS)						
Summary score	−0.017	(−0.08, 0.04)	−0.086	(−0.15, −0.03) **	0.174	(0.12, 0.23) ***
Posttraumatic Growth (PTGI)						
Summary score	0.243	(0.17, 0.31) ***	0.233	(0.16, 0.30) ***	0.166	(0.10, 0.24) ***
Relating to others	0.239	(0.17, 0.31) ***	0.230	(0.16, 0.30) ***	0.152	(0.08, 0.22) ***
New opportunities	0.167	(0.10, 0.24) ***	0.153	(0.08, 0.22) ***	0.146	(0.07, 0.22) ***
Personal strength	0.168	(0.10, 0.24) ***	0.146	(0.07, 0.22) ***	0.152	(0.08, 0.22) ***
Spiritual change	0.449	(0.39, 0.51) ***	0.502	(0.45, 0.55) ***	0.095	(0.02, 0.17) *
Understanding of life	0.169	(0.10, 0.24) ***	0.165	(0.09, 0.24) ***	0.109	(0.04, 0.18) **
SF-8 Health Survey						
Physical component summary (PCS)	−0.002	(−0.06, 0.06)	−0.115	(−0.18, −0.05) ***	0.263	(0.21, 0.32) ***
Mental component summary (MCS)	0.023	(−0.04, 0.08)	−0.095	(−0.16, −0.03) **	0.319	(0.26, 0.37) ***

Note: *** *p* < 0.001, ** *p* < 0.01, and * *p* < 0.05; CI = Confidence Interval.

**Table 4:** Descriptive characteristics of the sample and a nonparametric comparison of the values of the SWBS, RWB, and EWB (consisting of positively worded items only) in the sociodemographic groups.

		SWBS		RWB		EWB	
Groups	*n* (%)	M (SD)	Comparison of Groups	M (SD)	Comparison of Groups	M (SD)	Comparison of Groups
Total	1018 (100)	29.88 (7.66)		15.62 (6.23)		14.26 (3.03)	
Gender							
1. Male	496 (48.7)	28.51 (7.43)	*p* = 0.001	14.46 (6.11)	*p* < 0.001	14.05 (3.07)	*p* = 0.021
2. Female	522 (51.3)	31.19 (7.65)		16.72 (6.15)		14.47 (2.98)	
Age groups (years)							
1. 18–24	110 (10.8)	28.89 (7.35)	n.s.	14.45 (6.04)	*p* = 0.001 (1–6 *, 2–6 *, 3–6 *)	14.44 (3.35)	*p* = 0.002 (2–6 **, 3–6 *)
2. 25–34	187 (18.4)	29.74 (7.75)		15.02 (6.34)		14.72 (2.82)	
3. 35–44	199 (19.5)	29.33 (7.80)		14.75 (6.45)		14.58 (3.11)	
4. 45–54	166 (16.3)	29.94 (7.66)		15.74 (6.22)		14.20 (2.98)	
5. 55–64	168 (16.5)	30.33 (8.00)		16.42 (6.16)		13.91 (3.06)	
6. 65+	188 (18.5)	30.74 (7.27)		16.98 (5.77)		13.76 (2.87)	
Are you religious?							
Yes, I am a member of a church	380 (37.3)	34.39 (5.75)	*p* < 0.001 (1–2 ***, 1–3 ***, 1–4 ***, 2–3 ***, 2–4 ***, 3–4 *)	19.58 (3.92)	*p* < 0.001 (1–2 ***, 1–3 ***, 1–4 ***, 2–3 ***, 2–4 ***, 3–4 **)	14.81 (2.61)	*p* = 0.001 (1–2 *, 1–3 **)
Yes, not a member of a church	309 (30.4)	31.38 (6.19)		17.21 (4.34)		14.17 (2.83)	
No	249 (24.5)	23.97 (6.50)		10.35 (5.33)		13.62 (3.60)	
No, I am a convinced atheist	80 (7.9)	21.06 (4.40)		7.06 (4.04)		14.00 (3.24)	

Note: M = mean, SD = standard deviation; * *p* < 0.05, ** *p* < 0.01, and *** *p* < 0.001; n.s. = non-significant (*p* > 0.05); *p*-value belongs to the overall comparison of all groups, while the results in parentheses come from multiple group comparison.

## Data Availability

The data are available upon request.

## Abbreviations

The following abbreviations are used in this manuscript:

BRS	Brief Resilience Scale
CAPI	computer-assisted personal interviewing
CFA	confirmatory factor analysis
CFI	comparative fit index
CI	confidence interval
DWLS	diagonally weighted least squares
EWB	Existential Well-Being
MCS	mental component summary
PCS	physical component summary
PTGI	Post-traumatic Growth Inventory
RMSEA	root-mean-square error of approximation
RWB	Religious Well-Being
SRMR	standardized root mean square residual
SWBS	Spiritual Well-Being Scale
SWBS-Sk	shortened version of the Slovak SWBS scale consisting of seven positively worded items
TLI	Tucker–Lewis index
